# Circulating chemokine ligand levels before and after successful kidney transplantation

**DOI:** 10.1186/s12950-016-0141-4

**Published:** 2016-10-26

**Authors:** Hamdi Elmoselhi, Holly Mansell, Mahmoud Soliman, Ahmed Shoker

**Affiliations:** 1 St. Paul’s Hospital, Saskatchewan Renal Transplant Program, Saskatoon, SK Canada; 2 College of Pharmacy and Nutrition, University of Saskatchewan, Saskatchewan, Canada; 3 Division of Nephrology, Department of Medicine, University of Saskatchewan, University of Saskatchewan, 103 Hospital Drive, Saskatoon, SK S7N 0W8 Canada

**Keywords:** Chemokine, Inflammation, Kidney transplantation, Hemodialysis

## Abstract

**Background:**

Chemokine ligands (CCLs) play a pivotal role in tissue injury before and after kidney transplantation. Meanwhile, transplantation improves patient’s survival and diminishes morbidity. It is hypothesized, then, that kidney transplantation diminishes pre-transplant (pre-TX) levels of circulating inflammatory CCLs. This retrospective study compared circulating levels and profiles of CCLs before transplantation (pre-TX) and after transplantation (post-TX).

**Methods:**

Nineteen CCLs (1, 2, 3, 4, 5, 8, 11, 13, 15, 17, 21, 24, 26, 27, CXCL 5, 8, 10, 12 and 13) were measured in 47 stable post-TX recipients, and their stored pre-TX plasma was analyzed by multiplexed fluorescent bead-based immunoassay. Twenty normal controls were included for comparisons. Normalized data was presented as mean ± SD and non-normalized data as median (5–95 % CI). Significance was measured at p < 0.01. Arbitrary upper and lower margins for each CCL at the 95 % CI or 2SD levels in each group were chosen to calculate the percentile of patients in the other group who exceeded these limits. Significant CCL levels present in more than 75 % of patients in a group that exceeded the arbitrary upper or lower set margins in the other two groups were labeled as preferentially characteristic for the respective group.

**Results:**

More than 75 % of pre- and post-TX patients had levels that exceeded the upper control for CCL1, 11, 15 and CCL15, CCL26 and CXCL13 levels, respectively. More than 75 % of pre- and post-TX patients exceeded the lower control for CCL3, 21, and CCL5 limits, respectively. More than 75 % of post-TX patients demonstrated elevated levels of CCL2, 3, 21, 26 and CXCL13 above the upper pre-TX cut offs. Meanwhile, more than 75 % of post-TX patients exceeded the lower pre-TX levels for CCL1, 4, 5, 8, 13, 15, 17, 24 and CXCL8 and10. Pre-TX was preferentially characterized by elevated CCL1 and 15 and diminished CCL3 and 21. Post-TX was preferentially characterized by elevated CCL26 and CXCL13 and diminished CCL4 and 5.

**Conclusion:**

End stage kidney disease is associated with enhanced circulating inflammatory chemokine levels. Stable kidney transplantation is associated with 1) lowered burden of circulating inflammatory chemokine levels and, 2) elevation in the pro T-helper2 chemokine, CCL26 and the homeostatic CXCL13.

**Electronic supplementary material:**

The online version of this article (doi:10.1186/s12950-016-0141-4) contains supplementary material, which is available to authorized users.

## Background

Chemokines are small proinflammatory proteins that act as both chemoattractants and activators of leukocytes [1]. Chemokines are considered as a subset of the cytokine family responsible for cell migration, activation and tissue injury [2]. At least 50 structurally related chemokines have been discovered thus far, and they can be categorized into four subfamilies. An additional 20 receptors have been identified. On binding to their cell receptors, chemokine C-C motif ligands signal leukocyte recruitment and activation leading to tissue inflammation [3]. Extensive literature has delineated the pivotal role of CCLs in dialysis patients [4], vascular injury [5, 6] and transplant pathology [7].

Most chemokines share multiple cell receptors and have promiscuous functions ensuring robust inflammatory responses [1]. In general terms, however, the chemokines C-X-C motif ligand (CXCL) predominantly associates with neutrophil recruitment and activation [8], whereas the chemokines C-C motif ligand (CCL) is targeted more to lymphocyte and macrophage activation [8]. It is widely recognized that CCL14, 19, 20, 21, 25, 27, as well as CXCL12 and 13 have homeostatic functions [8], while typically CCL2, 3, 4, 5, 11, CXCL1, 2, 8, and 10 play a proinflammatory role [8]. Many inflammatory diseases and animal models demonstrate certain chemokine profiles with attracting T helper (Th) 1 or Th2 immunopathology. Th1 is typically associated with interferon (IFN) gamma, CCL1, 2, 8, 11, CCL3, CCL4, CCL5 and CXCL10 [9, 10] while Th2 is associated with IL4, IL5, IL13 and CCL3 [11]. The Th1 response is associated with a delayed type of hypersensitivity and cellular rejection [12], while Th2 is associated with humoral responses and tissue fibrosis [12]. Since certain chemokines are known to associate with certain biological functions, disruption of a particular chemokine activation cascade has been used as a promising tool to identify putative targets to treat human diseases [13–15]. A plethora of potential therapeutic agents exist that can block chemokine receptors and disrupt chemokine functions [16, 17]. It is a matter of time before the use of these agents in routine clinical practice will be achieved.

Hemodialysis patients awaiting transplantation have a high risk of mortality and morbidity. This is largely due to the presence of cardiovascular disease in which chemokines play a critical role [18]. After a successful renal transplant, the patient’s risk of having a cardiovascular events diminishes, but remains elevated compared to the general population. This is in part due to enhanced circulating chemokine ligand levels [19].

Therefore, studying chemokine biological functions before and after renal transplantation may assist in defining the magnitude of inflammatory burden caused by differentially elevated chemokine levels, and help to identify putative chemokines of potential future therapeutic importance in this population.

With this in mind, we aimed this study to compare the circulating chemokine profiles before and after kidney transplantation. We were interested in determining which circulating chemokine best distinguishes the impact of transplantation. We also sought to identify the chemokine which best correlates with the stable hemodialysis state in patients waiting for a transplant, as well as and the impact of transplantation thereof.

## Methods

The protocol was approved by the Regional Ethics Board at the University of Saskatchewan (Bio #11-220). Forty-seven patients were identified during the post-transplant period as eligible for the study based on the following inclusion criteria: had been stable on hemodialysis for at least 6 months, and had stored plasma immediately prior to transplant surgery at -80 °C. All renal transplant recipients (RTR) were adults older than18 years old and were followed in one out-patient university clinic. The following subjects were excluded from participation: those requiring hospitalization or have a change of more than 10 % in serum creatinine in the previous 3 months, treatment for any acute illness, apparent infection on the study visit and those who have a biopsy proven BK viral nephropathy. Written consent was taken from participants and plasma samples were collected for analysis of a large number of inflammatory chemokines including chemokine ligands. Patient demographics (age, sex, race), cause of kidney failure, mode of dialysis prior to transplant were collected. The estimated glomerular filtration rate (eGFR) was calculated from the following equation: GFR (CKD-EPI) =141X min(Scr/k,1)^α^ X max(Scr/k,1)^-1.209^ X.993^Age^ X1.018 [if female] X (1.159 [if black] where k = 0.7 if female, k = 0.9 if male, α = -0.329 if female, α = -0.411 if male, min = minimum of Scr/k or 1 and max = maximum Scr/k or 1, Scr = serum creatinine (mg/dL).

Blood was collected from healthy controls. Volunteers were excluded if they were smokers, were under treatment for any acute illness or had infection, hypertension, cancer, pregnancy, diabetes, or had experienced previous cardiovascular (CV) events. Mean age was 41 ± 14.1 years old and 60 % were females.

### Multiplexed fluorescent bead-based immunoassay

Plasma from 47 RTR and 20 normal controls was frozen at -80 °C until time of immunoassay (Luminex) measurement. The nineteen CCLs measured in this study were part of two luminex kits (Milliplex Map Human Cytokine/Chemokine Panel II 23-plex, EMD Millipore, Billerica, MA, USA and Bio-Plex Pro Human Cytokine 27-plex, Bio-Rad Laboratories Canada Ltd, Mississauga, Ontario, Canada). Assays were performed as per manufacturer instructions. Briefly, samples were centrifuged to remove the fat and particles, and added to the plate with the bead mixture. Incubation occurred for 30 min, and then washing was performed three times. Biotin-coupled antibody cocktail was added to each well, and the plate was incubated for 30 min followed by washing. Streptavidin conjugated phycoerythrin was added to the plate and incubated for 15 min in dark room. Finally assay buffer was added after washing, and the analysis was carried out on the Bio-Plex 200 instrument. The data was analyzed by Bio-Plex Manager Software (version 6.1). Concentrations obtained by the standard curve were expressed in pictograms per milliliters [20, 21]. Levels below the detection levels reported by the company, particularly in normal controls were assigned the lowest detection limit to enable us to perform the statistical comparisons.

### Statistics

SPSS version 22® (IBM Corp., Armonk, NY, USA) was used for the data collection and analysis. Data were calculated and presented as the mean ± standard deviation (SD) for normalized data and median (CI 95–5 %) for non-normalized data. All of the hypotheses tested were 2-tailed and a *p* value < 0.01 was considered statistically significant. Group differences were analyzed by Student *t* test and Mann Whitney for normally distributed, non-normally distributed variables, respectively.

Normality was assessed by Shapiro-Wilk test (because the data was less than 2000). Comparisons between chemokine ligand levels were performed between pre-TX and post-TX patients groups and between each patient group and control. Comparison results were presented in two ways. First, we presented the percentile of pre- and post-TX patients who had chemokine levels above or below an arbitrary defined upper and lower control CCL levels. For the sake of this study we set the lower control set point at the 5 % CI or 2SD below the median or mean control levels, respectively. Similarly, the upper control level was set to be at least 95 % CI or 2 SD above the median or mean control CCL level, respectively. The same method was used to calculate the percentile of post-TX patients with CCL levels above or below the pre-TX predefined levels.

## Results

Table 1 shows patient’s demographics. Forty-seven out of 65 patient participants were deemed stable and participated in the study. The most common reason for exclusion of the 18 other potential participants was that the patient was not scheduled for routine blood work on the same day of the clinic appointment, followed by the presence of flu-like symptoms or urinary tract infections. The average pre-transplant age was 42.7 years, and the average time post-transplant was 6.51 ± 2.03 years. The mean duration between the Pre-TX and Post-TX- test points was 9.82 (9.82–10.14) years. Approximately half (51.1 %) of the RTR were male. Most patients were on triple immunosuppressive drug therapy, with a mycophenolic acid derivative, a calcineurin inhibitor, and prednisone, and all patients were on hemodialysis prior to the transplant.

**Table 1:** Patient demographics

Parameter	Pre-TX	Post-TX	*P* value
Number	47	47	
Male (n) %	(24/47) 51.1 %		
Female (n) %	(23/47) 48.9 %		
Patient age	42.73 ± 14.71	49.11 ± 14.53	0.049
Patient age ≥ 60 (yrs) (n) %	12.80 %	25.30 %	
Ethnic origin			
Caucasian	72.34 %		
First Nation or Metis	25.53 %		
Asian	2.13 %		
Diabetic nephropathy	29.78 %		
Type of TX (LD/DD)	13/34		
Hemodialysis duration (yrs)	3.01 (1.72–4.13)	─	
Duration transplant (yrs)	─	6.51 ± 2.03	
Duration between first and second test (yrs)	9.82 (9.82–10.14)		
GFR (CKD-EPI)	─	52.1 (38.6–61.3)	
GFR (CKD-EPI) ≥ 60 (n %)	─	37.5 %	

The results are presented as mean ± SD for normally distributed data and median (confidence intervals 5–95 %) for non-normality distributed variables

*LD* living donor, *DD* deceased donor

A) **Comparison between pre-TX CCL and control levels**
  Results are presented in Table 2(a-b). Out of the 19 CCL measures, CXCL5, 8, 12 and 13 were similar to control levels. Nine CCLs (CCL1, 8, 11, 13, 15, 17, 24, 27, CXCL10) were significantly elevated and five CCLs (CCL2, 3, 5, 21, 26) were significantly lower than the control values.

**Table 2:** Comparison of circulating levels of inflammatory chemokine ligands in patient and control groups

a					
Chemokine (pg/m)	Control (*n* = 20)	Pre-transplant	Post-Transplant		
CCL1 (47/44)	2.9 (2.0–3.8)	7.7 (5.4–9.1)	2.0 (2.0–2.4)		
CCL2 (47/45)	231.0 ± 77.9	55.3 (38.9–74.4)	108.1 (98.3–144.5)		
CCL3 (46/44)	7.8 (5.3–9.0)	2.1 (1.8–2.5)	4.2 (3.7–4.7)		
CCL4 (46/44)	118.3 (96.2–127.2)	149.9 (128–177.8)	47.0 (34.1–80.7)		
CCL5 (46/41)	23805.2 ± 4535.9	12399.7 (8403.6–14469)	3312.5 (2474.2–5636.2)		
CCL8 (47/44)	27.8 ± 13.6	49.9 (41.4–56.7)	25.8 ± 1 0.6		
CCL11 (47/44)	39.0 (14.9–88.1)	192.4 (156.9–236.5)	80.8 (39.2–130.6)		
CCL13 (46/42)	44.0 ± 14.4	123.3 (100.2–148.5)	37.6 (20.1–57.5)		
CCL15 (47/40)	1292.0 (803.5–1453.7)	7192.7 (5758.8–9180.9)	3233.5 (2233.1–4466.5)		
CCL17 (46/43)	34.4 (25.0–52.4)	111.3 (65.2–162)	20.9 (16.1–32.3)		
CCL21 (46/44)	290.8 ± 80.3	37.9 (9.7–76.3)	183.7 (159.2–262.3)		
CCL24 (47/43)	494.7 (282.8–720.4)	836.1 (667.4–1396.1)	304.9 (223.1–420.9)		
CCL26 (47/44)	16.7 ± 10.1	3.7 (3.1–4.6)	48.8 (48.8–48.8)		
CCL27 (47/43)	424.9 ± 161.3	1328 (1038.7–1546.9)	816.9 ± 338.7		
CXCL5 (46/41)	511.4 (455.9–688.0)	339.7 (243.2–410.4)	337.9 (222.2–443.3)		
CXCL8 (47/41)	28.3 ± 8.7	25.7 (21.6–31.9)	11.4 (8.1–13.4)		
CXCL10 (46/44)	1183.0 ± 503.1	1868.8 (1601.6–2537.1)	726.8 (542.5–942.8)		
CXCL12 (47/44)	2424.7 (2011.8–2816.5)	1987.7 (1567.1–2255.8)	2220.8 (1400.6–2687.5)		
CXCL13 (46/44)	19.4 (13.1–23.5)	13.7 (9.9–18.8)	52.5 (33.1–60.0)		
b					
Chemokine (pg/m) # (pre-TX/post-TX)	∆ change	% change	P1	P2	P3
CCL1 (47/44)	-4.7 (-6.8 - -3.4)	-68.6 %	0.000	0.010	0.000
CCL2 (47/45)	64.1 (47.8–86.6)	113.9 %	0.000	0.000	0.000
CCL3 (46/44)	2.1 (0.8–2.6)	90.8 %	0.000	0.000	0.000
CCL4 (46/44)	-98.2 (-122.9 - -67.2)	-70.5 %	0.010	0.000	0.000
CCL5 (46/41)	-814.7 ± 9480.3	-75.6 %	0.000	0.000	0.000
CCL8 (47/44)	-23.7 (-29.6 - -15.8)	-47.9 %	0.000	0.720	0.000
CCL11 (47/44)	-97.4 (-164.4 - -61.59)	-59.8 %	0.000	0.080	0.000
CCL13 (46/42)	-72.2 ± 77.1	-63.5 %	0.000	0.720	0.000
CCL15 (47/40)	-3459.1 (-4848.2 - -1000.7)	-50.6 %	0.000	0.000	0.000
CCL17 (46/43)	-89.7 (-119.1 - -50.1)	-77.6 %	0.000	0.020	0.000
CCL21 (46/44)	157.6 (65.1-208.2)	404.0 %	0.000	0.120	0.000
CCL24 (47/43)	-660.1 (-830.3--535.5)	-71.0 %	0.000	0.010	0.000
CCL26 (47/44)	44.4 (42.8–45.8)	1096.0 %	0.000	0.000	0.000
CCL27 (47/43)	-575.7 (-844.3 - -238.7)	-40.3 %	0.000	0.000	0.000
CXCL5 (46/41)	-49.1 (-218.3–41.0)	-18.8 %	0.030	0.010	0.690
CXCL8 (47/41)	-10.8 (-22.7 - -7.8)	-54.9 %	0.720	0.000	0.000
CXCL10 (46/44)	-1015.3 (-1423.6 - -731.4)	-64.2 %	0.000	0.040	0.000
CXCL12 (47/44)	133.3 (-571.7–855.5)	4.9 %	0.050	0.210	0.630
CXCL13 (46/44)	34.1 (16.8–46.1)	213.5 %	0.080	0.000	0.000

For both tables a and b, the results are presented as mean ± SD for normally-distributed, and median (5–95 %) confidence intervals for non-normality distributed data, respectively. Sample size for patients in the pre-TX/post-TX groups are presented between brackets in column 1. Sample size for the control was *n* = 20 for all chemokines

Delta change is calculated from: (post-TX chemokine level—pre-TX chemokine level). Percentage change in inflammatory chemokines level is calculated from: (post-TX chemokine level—pre-TX chemokine level))/pre-TX chemokine level. P1 represents the *p*-value between control and pre-TX; P2 represents the *p*-value between control and post-TX; P3 represents the *p*-value between pre-TX and post-TX. *P*-value computed by Mann-Whitney u – test
B) **Comparison between post-TX CCL and control levels**
  As presented in Table 2(a-b), seven chemokines had normal levels. They were: CCL8, 11, 13, 17, 21, CXCL 10 and 12. Only four markers (CCL15, 26, 27 and CXCL 13) were significantly elevated and the remaining eight markers (CCL1, 2, 3, 4, 5, 24, CXCL5, 8) were significantly lower than the control values.
C) **Similarities and dissimilarities between CCL levels in both patient’s groups (pre- and post-TX) compared to controls**
  Both of the pre- and post-TX patients shared some similarities when compared to the control group. In particular, CCL2, 3, 4 and 5 were significantly lower in both groups when compared to control CCL levels. Both patients’ groups had CCL levels above the control group for CCL 15 and CCL 27 only.
  After transplantation, four pre-TX CCL (CCL 8, 11, 13 and CXCL10) levels were elevated above control values and were normalized after transplantation. Similarly, two significantly diminished Pre-TX CCL levels (CCL17 and 21) saw normalization after transplantation.
  On the other hand, CXCL13 was the only chemokine that had normal level before transplantation and saw a significant increase post-transplantation. Of note, CCL 26 was the only marker that was significantly diminished before transplantation and saw a significant elevation after transplantation. Pre transplant CCL1, 2 and 24 levels were significantly elevated above control levels and were decreased after transplantation to reach a significantly lower level when compared to control values. On the other hand, significantly diminished pre-TX CCL 17 and 21 levels were normalized in the post-TX group.
D) **Comparison between pre- and post-TX CCL levels**
  CXCL5 and 12 had similar levels before and after transplant, while thirteen markers (CCL1, 4, 5, 8, 11, 13, 15, 17, 24, 27 and CXCL5, 8, 10) saw a significant decrease, and only five markers (CCL2, 3, 21, 26 and CXCL13) increased after transplantation. Table 2 shows also the delta (∆) changes in CCL levels after transplantation. Maximum delta changes were seen CCL15 and CXCL10.
E) **Percentiles of pre- and post- TX patients with chemokine levels exceeding the control 95 % CI levels**
  More than 75 % of pre-TX patients had levels below the lower cut off for CCL3, 21 and levels above the set upper level for CCL1, 11, 15. After transplantation more than 75 % had lower levels for CCL3, 4, 5 and higher levels for CCL15, 26 and CXCL13. The cumulative percent of pre- and post-TX patients with chemokine levels below the set lower control limit was 13 % (2.1–66 %) and 35.3 ± 29.6 %, respectively. The percent of pre- and post-TX patients with levels above the 95 % CI control values were 23.9 % (4.3–69.6 %) and 11.4 % (4.5–34.1 %), respectively. More information can be viewed in the Additional file 1: Table S1.
F) **Percentiles of post-TX patients with chemokine levels exceeding the pre-TX 95 % CI levels**
  More than 75 % of post -TX patients had lower levels for CCL1, 4, 5, 8, 13, 15, 17, 24, CXCL 8 and 10, compared to the pre-TX. Meanwhile, over 75 % of patients had levels above the upper pre-TX levels for CCL2, 3, 21, 26 and CXCL13. The cumulative percent of post-TX patients with chemokine levels below the set lower pre-TX limit was 81.8 % (4.5–92.7 %). The percent of post-TX patients with levels above the 95 % CI control values were 7.3 % (2.4–98.1 %). For more information see Additional file 1: Table S2.

### Circulating chemokine profiles preferentially characteristic for patients before and after TX

Pre-TX CCLs level that showed significance and exceeded, as well, the upper cut off levels in post-TX and control values in at least 75 % of patients were CCL1 and 15. CCLs in the same group that exceeded the lower cut-offs in the other two groups in >75 % of pre-TX patients were CCL3 and 21. Likewise the post-TX CCLs that exceeded both the pre-TX and control levels in over 75 % of post- TX patients were CCL26 and CXCL13 while CCL 4 and 5 were diminished in more than 75 % of post-TX patients below the control and pre-TX cut off levels.

## Discussion

The results raise several points for discussion. In general, statistical analysis may not reflect robust biological differences. In addition, chemokine levels vary significantly even among normal people [22]. To strengthen the biological significance of our results we opted to define arbitrary stringent CCL cut-off levels in the comparative analyses, in addition to the conventional statistical methods. We enumerated the number of patients who had levels beyond the 95 % CI and calculated the percentage of patients exceeding these limits. We also set the *P* value at <0.01 to perhaps strengthen our conclusions. Secondly, the literature is replete of information on the inflammatory and homeostatic nature of chemokines in health and disease [23]. We will first comment on CCL profiles before and after transplant and then focus on CCLs that preferentially associate with patients before and after transplantation.

The results of the pre-TX CCL levels raise several points. Among the 19 CCL chemokines tested, only CXCL8 which plays a role in neutrophil trafficking [24] and CXCL13 which is significant in B and T cell trafficking in lymphoid tissues, were within the normal range. Typically, CXCL8 has an inflammatory function [25] and CXCL13 has a homeostatic function [26]. Of interest, in the conventional non-parametric analysis, the proinflammatory CCL1, 4, 8, 11, 13, 15, 24, CXCL10 and the homeostatic CCL27 [8] were significantly elevated above controls. Remarkably, more than 75 % in this group had CCL1, 11, and 15 levels exceeding the upper control set limits. Chemokines that qualified to be preferentially characteristic to pre-TX were CCL1 and 15. That is because their levels were significantly higher than the other two groups and more than 75 % of the pre-TX patients demonstrated levels above the upper 95 %CI in both the control and the post-TX margins. CCL1and CCL15 have strong proinflammatory properties [27]. These results are supportive of the reported evidence of increased Th1 in patients with atherosclerosis [28] and in dialysis patients [27]. This work defines the elevated proinflammatory chemokines in stable dialysis CKD stage 5 patients and highlights the importance of CCL1 and 15 as central in this process.

In the same pre-TX group, the proinflammatory CCL2, 3, and 5, and the homeostatic CCL21 levels was lower than both control and post-TX patients. Again, CCL3 and 21 districted themselves as the two chemokines that were preferentially and characteristically diminished in more than 75 % of pre-TX patients below the control and post-TX 95 %CI lower limit. CCL3 is important for T cell and monocyte trafficking [29] and a key function of CCL21 is in T cell and DC homing to lymph nodes [30], which explains in part the role of deficient chemokines in the immune deficiency state of CKD stage 5.

The profile of chemokine levels post-transplantation requires several comments. First, CXCL5 and 12 had normal levels. CCL15, 26, 27 and CXCL13 were significantly elevated above control levels. Exceedingly high chemokine levels that were preferentially characteristic of post-TX were CCL26 and CXCL13. CCL26 has proinflammatory properties and plays an important role in Th2 responses [31]. CXCL13 has a homeostatic function and a key role in T and B cell trafficking in lymph nodes [32]. In contrast, CCL4 and 5 were the preferentially characterized post-TX chemokines that exceeded the lower 95 %CI margins of both the control and pre-TX patients. CCL4 is key in T/DC interaction [33], while CCL5 is key for innate and adaptive immunity [34]. Calcineurin inhibitor therapy used to suppress the immune system is associated with a bias toward Th2/Th1 at least in liver transplant recipients [20].

Further discussion on some of the similarities in chemokine levels pre- and post-TX shows interesting findings. First, CXCL12 had normal level in both groups. Secondly, both patient groups had diminished levels of CCL 2, 3, and 5. CCL2 key function is innate immunity and Th2 responses. CCL5 key function includes innate immunity and adoptive immunity [34].

Preferentially increased pre-TX CCL levels included CCL1 and 15, while preferentially diminished pre-TX CCL levels were CCL3 and 21. Pre-TX CCL15 was elevated above control to the extent that more than 90 % of patients in either patient’s group achieved level above the upper 95 % CI. Further, pre-TX CL15 was so exceedingly high to the extent that 93 % of pre-TX had levels above the 95 % CI post-TX target. As such we qualified CCL15 as preferentially characteristic of patients before TX. The significantly elevated CCL15 level confirms its role in tissue injury in patients on dialysis and patients with impaired native kidney function [21]. CCL15 is a potent angiogenic factor [35] that contributes to chronic inflammation and fibrosis [36]. It is elevated in patients with progressive CKD [37]. Elevated CCL15 may target the native kidney as a source of inflammatory reservoir and also aggravate graft dysfunction. A striking recent report describes how cleavage of CCL15, but not other CCL family members, leads to more active form [38]. Elevated CCL15 may aggravate graft dysfunction. The results raise its potential as a therapeutic target before and after transplantation. CCL1 has a proinflammatory function and supports Th2 responses [39]. In contrast to CCL15, a mirror image finding is CCL3. It distinguishes itself as the only diminished chemokine in both patient’s groups and which more than 75 % of pre and post-TX patients had levels below the lower set control margin. In addition it’s pre-TX level was too low that 79.5 % of post-TX patients have levels exceeding the upper pre-TX cut off. Thus diminished CCL3 was qualified as characteristic of pre-TX. It binds to CCR1 and 3. CCL3 has a proinflammatory function and plays a role in T cell trafficking [8, 30]. Key function of CCL3 is innate immunity and T-cell and monocyte trafficking [29]. Decreased level of CCL21 was also preferentially characteristic of pre-TX. CCL21 key function is T cell and DC homing to lymph nodes [30].

Preferentially elevated post-TX CCLs included CCL26 and CXCL13 while CCL4 and 5 had preferentially diminished levels. CCL26 supports Th2 responses and CXCL13 is important for T and B cell trafficking. CCL26 was unique in that its level was significantly diminished before TX and saw a significant elevation in a large number of patients after transplant to the extent that in all post-TX patients, CCL26 was uniquely elevated above the upper pre-TX level. This central finding suggests that there may be a biological relevance to this molecule in transplant immunobiology [40, 41]. Interestingly, this chemokine uses-receptor that is present on eosinophils, basophils, T helper 2, monocytes, dentritic cells, plasma cells and natural killer cells.

Transplantation seems to normalize some higher chemokine levels in CKD stage 5 which included in this work CCL8, 11, 13, and CXCL10. These are predominantly inflammatory chemokines [11]. Of interest, transplantation seemed to even suppress previously elevated chemokines in the hemodialysis patients. For example, CCL1 was significantly elevated before transplantation and almost 90 % of pre-TX patients had higher level above the control compared to only 11 % after transplantation. CCL1 levels dropped below the lower control set limit in more than half the TX patients. CCL4, and 24 had a similar fate. In contrast, transplantation did not seem to improve diminished levels of certain chemokines like CCL3, which is important for T cell and monocyte trafficking [29], and CCL5, which is important in innate and adoptive immunity [34].

The mean percentiles of pre-TX patients with chemokine levels exceeding control levels (23.9 %) were almost doubled compared to after transplantation (11.4 %). Conversely the mean percentiles of pre-TX patients with diminished CCL levels below the lower control was 13 % before transplant and increased to 35.5 % after transplantation, confirming the relatively diminished inflammatory burden after transplant. There was also a noticeable shift in chemokine profile that favors Th1 responses to Th2 responses after transplantation.

A major impetus for studying the chemokine system is the possibility of using a chemokine blockade to treat inflammation induced tissue injury as seen in CKD and even after transplantation and therefore, we believe that this work has clinical relevance. Uremic toxins can prime peripheral polymorphonuclear leukocyte as a key mediator of low grade inflammation [42]. In fact persistent inflammation has been suggested as a catalyst for other risk factors in chronic kidney disease [43]. CVD is the leading cause of morbidity in dialysis patients and after organ transplantation. CCL15 is a chemokine that exerts its biological activities through CCR1 and CCR3. A link between increased its plasma levels and vascular injury and progressive CKD has been suggested by several researchers based on the strong association between increased chemokine levels and progressive renal disease [44, 45]. There are clinical and preclinical studies using chemokine agonists and antagonists in patients with CKD [46, 47]. For example, CCL15 binds to CCR1. Blocking CCR1 with specific small molecule antagonists was shown to retard progression in various types of rodent CKD models [48]. As such we identify CCL15 as a target before and after transplantation. We also identified CCL26 as a potential target after transplant. In addition we identified a possible target for agonistic activity to improve immunity in the kidney patient such as CCL3, 4 and 5.

There are obvious limitations to this work, in addition to its retrospective nature and small number of patients. We note that the cross-sectional study design does not permit evaluating the association of biomarkers to incident of disease risk. We did not have kidney biopsies to correlate changes in chemokines with tissue pathology. We do not have a longitudinal follow up study of possible natural variation in circulating chemokine levels and did not have other groups of patients with renal failure induced tissue injury like cardiovascular diseases to prove causality between events and chemokine levels. The time between transplant study time and transplant dates are varied among patients. The number of controls was lower than those of transplanted subjects, moreover patients and controls were not sex matched (60 % of male in control group and about 50 % in the patients). Furthermore, there was some missing data due to insufficient blood samples on these individuals. In addition, measurements of these inflammatory markers in the same patient before and after transplantation should remain meaningful within the context of this study design. We plan further studies to address these clinically relevant questions.

## Conclusions

To conclude, the goal of this study was to identify circulating inflammatory chemokines of significance before and after transplantation in a stable RTR population. The results identified chemokines that preferentially characterized pre-TX, including CCL1, and 15 which exceeded the upper control and post-TX margins and CCL3 and 21 which exceeded the lower margin. After transplantation, CCL26 and CXCL13 exceeded the control and pre-TX margins, while CCL4 and 5 exceeded the lower set margins. Elevated circulating CCL 26 and CXCL13 seem to strongly associate with transplantation and are possible therapeutic targets to improve the post-TX course.

## Additional file

Additional file 1: Table S1. Percentage of patients with chemokine levels exceeding the control 5 % or 95 % CI values. **Table S2** Percentages of transplant patients with chemokine levels exceeding the pre-TX 5 % or 95 % CI values. (DOCX 133 kb)

## Abbreviations

CCLs: Chemokine (C-C motif) ligand
CXCLs: Chemokine (C-X-C motif) ligand
eGFR: estimated Glomerular Filtration Rate
Post-TX: Post-transplant
Pre-TX: Pre-transplant
TX: Transplant
