# Supplementary material for: Circulating chemokine ligand levels before and after successful kidney transplantation
Source: J Inflamm (Lond). 2016 Oct 26;13:32. doi: 10.1186/s12950-016-0141-4 (PMC5081672; doi:10.1186/s12950-016-0141-4)
Supplement: Additional file 1: Table S1. — Percentage of patients with chemokine levels exceeding the control 5 % or 95 % CI values. Table S2 Percentages of transplant patients with chemokine levels exceeding the pre-TX 5 % or 95 % CI values. (DOCX 133 kb) [file 12950_2016_141_MOESM1_ESM.docx]

**Supplementary Table 1:**

Percentage of patients with chemokine levels exceeding the control 5% or 95% CI values

| Chemokines (pg/ML) | Lower CI control Value | Upper CI control value | % of Pre-TX patients with values below lower CI control | % of Pre- TX patients with values above upper CI control | % of post-TX patients with values below lower CI control | % of post-TX patients with values above upper CI control |
| --- | --- | --- | --- | --- | --- | --- |
| CCL1 | 1.98 | 3.81 | 2.10% | 89.40% | 56.80% | 11.40% |
| CCL2 | 75.16 | 539.68 | 66.00% | 0.00% | 13.30% | 0.00% |
| CCL3 | 5.27 | 9.02 | 93.50% | 4.30% | 75.00% | 4.50% |
| CCL4 | 96.2 | 127.21 | 15.20% | 69.60% | 77.30% | 9.10% |
| CCL5 | 14733.37 | 53278.23 | 67.40% | 2.20% | 92.70% | 0.00% |
| CCL8 | 0.61 | 67.02 | 0.00% | 10.60% | 0.00% | 0.00% |
| CCL11 | 14.93 | 88.14 | 0.00% | 91.50% | 20.50% | 43.20% |
| CCL13 | 15.28 | 104.66 | 17.40% | 63.00% | 33.30% | 11.90% |
| CCL15 | 803.46 | 1453.67 | 4.30% | 93.60% | 2.50% | 90.00% |
| CCL17 | 25.01 | 52.37 | 13.00% | 73.90% | 55.80% | 9.30% |
| CCL21 | 130.14 | 678.21 | 80.40% | 0.00% | 31.80% | 18.20% |
| CCL24 | 282.77 | 720.4 | 6.40% | 59.60% | 46.50% | 4.70% |
| CCL26 | 0.83 | 45.04 | 0.0% | 4.30% | 0.00% | 84.10% |
| CCL27 | 102.41 | 1045.24 | 2.10% | 63.80% | 0.00% | 25.60% |
| CXCL5 | 455.92 | 688.03 | 69.60% | 23.90% | 68.30% | 14.60% |
| CXCL8 | 10.86 | 63.78 | 2.10% | 12.80% | 46.30% | 0.00% |
| CXCL10 | 176.74 | 3024.44 | 0.00% | 21.70% | 4.50% | 11.40% |
| CXCL12 | 2011.84 | 2816.54 | 53.20% | 21.30% | 45.50% | 34.10% |
| CXCL13 | 13.09 | 23.54 | 45.70% | 23.90% | 0.00% | 81.80% |
| Mean ±SD or median (5%-95% C.I) |  |  | 13.0 %( 2.1% - 66%) | 23.9 %( 4.3% - 69.6%) | 35.3%±29.6% | 11.4 %( 4.5% - 34.1%) |

Lower and upper control values were arbitrary set at 5% and 95% confidence interval (CI) values for non-normalized control values or two standard deviations (SD) below and above mean values for normalized data.

**Supplementary table #2:**

Percentages of transplant patients with chemokine levels exceeding the pre-TX 5% or 95% CI values

| chemokines (pg/mL) | Pre-TX lower CI value | Pre-TX upper CI value | % of post-TX patients with values below lower CI pre-TX | % of post-TX patients with vales above upper CI pre-TX |
| --- | --- | --- | --- | --- |
| CCL1 | 5.37 | 9.07 | 97.70% | 0.00% |
| CCL2 | 38.93 | 74.39 | 2.20% | 86.70% |
| CCL3 | 1.82 | 2.5 | 4.50% | 79.50% |
| CCL4 | 127.97 | 177.83 | 90.90% | 4.50% |
| CCL5 | 8403.63 | 14468.98 | 82.90% | 7.30% |
| CCL8 | 41.43 | 56.746 | 95.50% | 0.00% |
| CCL11 | 156.9 | 236.47 | 65.90% | 13.60% |
| CCL13 | 100.21 | 148.5 | 88.10% | 2.40% |
| CCL15 | 5758.8 | 9180.92 | 82.50% | 5.00% |
| CCL17 | 65.22 | 161.95 | 93.00% | 0.00% |
| CCL21 | 9.7 | 76.33 | 0.00% | 77.30% |
| CCL24 | 667.37 | 1396.05 | 95.30% | 0.00% |
| CCL26 | 3.062 | 4.61 | 0.00% | 100.00% |
| CCL27 | 1038.68 | 1546.93 | 72.10% | 4.70% |
| CXCL5 | 243.21 | 410.36 | 34.10% | 36.60% |
| CXCL8 | 21.57 | 31.92 | 92.70% | 4.90% |
| CXCL10 | 1601.61 | 2537.12 | 81.80% | 11.40% |
| CXCL12 | 1567.08 | 2255.84 | 36.40% | 50.00% |
| CXCL13 | 9.86 | 18.77 | 0.00% | 86.40% |
| Mean ±SD or median (5% - 95% CI) |  |  | 81.8 % (4.5% - 92.7%) | 7.3 % (2.4% - 98.1%) |

Set lower and upper levels in pre-TX patients were set at 5% and 95% confidence intervals (CI) for non-normalized values or two standard deviations (SD) below and above mean for normalized data.
